# Efficacy and safety evaluation of massage combined with traction for lumbar disc herniation: a meta-analysis

**DOI:** 10.3389/fmed.2026.1762760

**Published:** 2026-03-12

**Authors:** Baoli Zhao, Qi Zhao, Jing Yin, Hongdong Wang, Xiaohong Wu, Liyong Liu, Gang Bai, Junjie Li

**Affiliations:** 1 Department of TCM Orthopedics, Beijing Electric Power Hospital of State Grid Corporation of China, Beijing, China; 2 Department of TCM, Aerospace Center Hospital, Beijing, China; 3 Department of TCM, Beijing Electric Power Hospital of State Grid Corporation of China, Beijing, China

**Keywords:** lumbar disc herniation, massage, meta-analysis, traction, tuina

## Abstract

**Objective:**

To systematically evaluate, through a meta-analysis, the effectiveness and safety of massage combined with traction for the treatment of lumbar disc herniation (LDH).

**Methods:**

A comprehensive literature search was conducted across multiple Chinese and international databases, including CNKI, Wanfang, VIP (Chinese Science and Technology Journals Database), China Biomedicine (CBM), PubMed, Web of Science, and the Cochrane Library, covering the period from January 2020 to December 2024. Heterogeneity was assessed by the *Q* test and *I*^2^ statistic, and pooled ORs were calculated using fixed- or random-effects models. Sensitivity analysis and funnel plots were used to assess result stability and publication bias.

**Results:**

Twelve studies met the inclusion criteria. Meta-analysis showed that tuina combined with traction significantly improved clinical outcomes compared with control interventions, including JOA scores (OR = 4.56, 95% CI: 2.76–6.36, *p* < 0.00001), VAS scores (OR = −1.47, 95% CI: −2.40 to −0.55, *p* = 0.0002), ODI scores (OR = −12.73, 95% CI: −23.88 to −1.59, *p* = 0.03), and lumbar posterior extension scores (exploratory results based on two studies) (OR = 6.51, 95% CI: 3.57–9.46, *p* < 0.0001). No significant difference was observed for forward bending motion scores. Sensitivity analysis confirmed the robustness of the pooled estimates, while funnel plots suggested potential publication bias.

**Conclusion:**

This meta-analysis indicates that tuina combined with lumbar traction effectively improves lumbar function and reduces pain severity in patients with lumbar disc herniation.

## Introduction

Lumbar disc herniation (LDH) is a common pathological condition characterized by low back pain and/or radicular symptoms resulting from the displacement of the nucleus pulposus within the intervertebral disc, which may cause nerve root irritation or compression (1, 2). This condition is multifactorial in nature and may be associated with age-related degenerative changes, traumatic events, spinal instability or subluxation, reduced activity of deep stabilizing muscles, and decreased physical activity, rather than being attributable to degeneration alone. LDH presents a significant clinical challenge, with an incidence rate in China ranging from 8 to 25%, predominantly affecting young adults aged 20 to 50 years, with a higher prevalence among males (3). The intervertebral spaces L4–5 and L5–S1 are most frequently involved. Patients with LDH typically experience low back pain with or without radicular symptoms, as well as functional or muscular fatigue. In more severe cases, pain-related functional (antalgic) scoliosis, disuse-associated muscle atrophy related to impaired spinal stabilization, and reduced lumbar mobility may occur, thereby severely compromising quality of life (4).

With the advancement of the social economy, the incidence of LDH has progressively increased, particularly among younger populations (5–7). This rising prevalence underscores the importance of effective treatment options (8). Currently, LDH treatment can be broadly classified into conservative (non-surgical) and surgical approaches. Surgical intervention is necessary for only 10 to 20% of LDH patients, while more than 80% can benefit from non-surgical management. Common conservative options include physical therapy and pharmacotherapy, with lumbar traction and various forms of traditional Chinese medicine (TCM) frequently used as adjunctive rehabilitation approaches (9).

Lumbar traction is a widely used clinical rehabilitation technique, and recent randomized controlled evidence suggests that manual traction can alleviate lumbosacral spine pain associated with radicular symptoms (10). This therapeutic approach involves applying stretching and reverse stretching tension to the lumbar spine, specifically targeting intervertebral spaces such as L3, L4, L5, and S1. In addition to effects on spinal segments, traction may also modulate soft-tissue tension by stimulating proprioceptors and mechanoreceptors in muscles and fascia, thereby eliciting neuromuscular responses that promote relaxation. The primary goal of lumbar traction is to reduce nerve root irritation and related symptoms by increasing intervertebral space and decreasing mechanical compression, potentially facilitating partial retraction of the protruded nucleus pulposus. When combined with tuina (therapeutic massage), the intervention may further improve soft-tissue tension and local circulation (11). When combined with massage therapy, this integrated intervention can effectively reduce intervertebral disc pressure, relieve nerve compression, and enhance local circulation. As a result, it improves conditions such as congestion, edema, and adhesion, while promoting soft tissue repair through reduced inflammatory stimulation and providing analgesic effects. However, although prior systematic reviews and network meta-analyses have evaluated TCM-related conservative therapies for LDH, their evidence is limited for clarifying the incremental benefit of tuina plus traction specifically. Existing meta-analyses often pooled heterogeneous interventions (e.g., acupuncture-centered protocols, mixed manual therapies, or multimodal regimens) and diverse comparators, which makes it difficult to isolate the effect attributable to adding tuina to traction. In addition, key details such as traction modality/parameters and tuina technique/dosage, as well as adverse-event reporting, were frequently insufficient or inconsistent, further weakening the interpretability and clinical translatability of pooled estimates. Therefore, despite increasing clinical use, the comparative effectiveness and safety of tuina combined with lumbar traction versus traction alone remain uncertain and warrant a focused quantitative synthesis.

In recent years, traditional Chinese medicine (TCM) has gained increasing attention as a viable non-surgical treatment for LDH (12). This growing attention has been particularly evident in China, where TCM-based conservative and rehabilitative approaches are widely used in clinical practice, and it has also been increasingly discussed in broader clinical research contexts. However, the current evidence remains evolving, and reported benefits vary across modalities and study designs.

However, tuina, a traditional therapeutic massage for musculoskeletal disorders, including LDH, has received comparatively less attention in scientific literature, despite its promise as an external treatment for LDH. It is hypothesized that combining tuina with lumbar traction can enhance therapeutic outcomes by leveraging the benefits of both methods (5, 13). Given the growing interest in non-surgical treatments and the need for more robust evidence on the efficacy of TCM approaches, this meta-analysis was conducted to evaluate the safety and efficacy of tuina combined with traction in treating LDH.

## Materials and methods

### Material sources and retrieval strategies

We conducted a comprehensive literature search across multiple databases, including CNKI, Wanfang, WiP Chinese Science and Technology Journals, China Biomedicine, PubMed, Web of Science, and the Cochrane Library, covering the period from January 2020 to December 2024. Our Chinese search strategy utilized terms such as “TCM massage,” “lumbar traction,” and “lumbar disc herniation,” while the English search strategy employed MeSH terms like “Massage,” “lumbar traction,” and “lumbar disc herniation.” To ensure full reproducibility, we provide the complete PubMed search strategy as follows: (“Hernia, Intervertebral Disc”[Mesh] OR “lumbar disc herniation” OR LDH OR “intervertebral disc herniation” OR “disc protrusion”) AND (“Traction”[Mesh] OR “lumbar traction” OR “spinal traction” OR “mechanical traction” OR “manual traction”) AND (“Massage”[Mesh] OR tuina OR “Tui Na” OR “Chinese massage” OR “manual therapy”) AND (randomized controlled trial[Publication Type] OR randomized[Title/Abstract] OR randomly[Title/Abstract] OR trial[Title/Abstract]) AND (“2020/01/01”[Date - Publication]: “2024/12/31”[Date - Publication]). In addition to disease-specific terms, broader clinically relevant keywords were incorporated to enhance search sensitivity, including “low back pain” and related pain descriptors. Both manual and mechanical forms of lumbar traction were considered eligible interventions, and search terms such as “manual traction” and “mechanical traction” were included accordingly. This systematic review was designed to identify relevant studies that evaluate the effectiveness and safety of combining TCM massage with lumbar traction for the treatment of lumbar disc herniation.

### Inclusion and exclusion criteria

Literature inclusion criteria (only fully published, peer-reviewed journal articles with accessible full texts were considered eligible; clinical practice guidelines/consensus documents cited within included trials were not treated as eligible studies and were not included as separate records). (1) The studies included must involve patients who have been clinically diagnosed with lumbar disc herniation, with confirmation through imaging or other diagnostic criteria. Although lumbar disc herniation was used as a diagnostic inclusion criterion, this review specifically focused on patients with LDH accompanied by low back pain and/or radicular symptoms, as reflected by pain-related outcome measures such as the Visual Analogue Scale (VAS). (2) The study design should be a randomized controlled trial (RCT), which is widely recognized as the gold standard for evaluating treatment efficacy. (3) The outcome measures should include at least one of the following: clinical efficacy, Japanese Orthopaedic Association (JOA) score, Visual Analogue Scale (VAS) for pain, Oswestry Disability Index (ODI) for lumbar function, or lumbar motion score. These indicators are essential for assessing both the clinical and functional outcomes of the interventions. (4) The study should compare outcomes between two groups: an experimental group receiving massage combined with lumbar traction and a control group receiving only lumbar traction. If additional treatments were administered to either group, they must have been applied consistently and uniformly to avoid introducing bias.

Exclusion criteria: (1) Duplicate studies, irrelevant literature, and review articles were excluded to ensure that only original research contributing new data was considered. (2) Studies based on animal models were excluded, as this meta-analysis focuses on clinical outcomes in human patients with lumbar disc herniation. (3) Studies were excluded if their outcome indicators did not align with the predefined inclusion criteria, such as those lacking clinical efficacy measures or using alternative scoring systems not comparable to JOA, VAS, or ODI. (4) Studies that did not include both a trial group (receiving massage combined with traction) and a control group (receiving traction alone) were excluded to ensure that the research directly addressed the question of interest. (5) Research with missing, incomplete, or unusable data was excluded, including studies where outcome data were not fully reported or contained obvious errors, potentially compromising the validity and reliability of the meta-analysis. (6) Studies published before 2020 were excluded to focus on the most recent and relevant research, ensuring that the analysis reflects current clinical practices and understanding of the treatment efficacy for lumbar disc herniation. (7) Additionally, studies were excluded if they were published in non-peer-reviewed sources or if their methodology did not meet standard quality criteria for RCTs, such as lacking appropriate randomization, blinding, or having a high risk of bias.

### Literature screening and data extraction

Two reviewers independently selected the studies. In cases of disagreement, third-party experts were consulted to resolve the differences. For the studies that met the inclusion criteria, data were extracted using a pre-established literature feature table. The extracted information included the research design type, sample size, and outcome indicators. Specifically, we collected details such as the study’s first author, publication date, and the intervention methods used in both the experimental and control groups.

### Risk of bias assessment

The quality of the included randomized controlled trials (RCTs) was assessed using the Cochrane risk of bias (RoB) tool, which evaluates several key areas to determine the potential impact of bias on study results. First, we assessed selection bias by evaluating the randomization procedures and allocation concealment. Next, we examined performance bias by ensuring that both participants and researchers were blinded to treatment assignments. Detection bias was also considered, with outcome assessors remaining unaware of the participants’ group allocations. Attrition bias was addressed by reviewing data handling methods and ensuring that all participants were accounted for in the analysis. Reporting bias was assessed by comparing the reported outcomes with those specified in the study protocol. Finally, other potential sources of bias, such as differences in baseline characteristics or deviations from the study protocol, were thoroughly scrutinized. Each study’s risk of bias in these domains was rated to provide a comprehensive assessment of its reliability.

### Sensitivity analysis

The studies that significantly influenced the combined effect size were identified and systematically excluded for further analysis. Each of these influential studies was individually reviewed by analyzing the forest plots generated from the initial analyses to assess their impact on the overall results. Following this, a meta-analysis was rerun to calculate new combined effect sizes (ORs and 95% CIs) and generate new forest plots for visual comparison before and after exclusion. Heterogeneity was re-evaluated using *Q*-tests and *I*^2^ statistics to assess any changes resulting from the exclusion of these studies. Finally, the sensitivity analysis results were compared with those from the initial meta-analyses, confirming that the exclusion of heavily weighted studies did not significantly affect the stability or reliability of the combined effect sizes, thereby validating the robustness of the meta-analysis findings.

### Statistical methods

NoteExpress 3.9 software was used for literature management, and Excel 2023 was employed for data collection and extraction. Statistical analysis was performed using RevMan 5.4.1. The chi-square (*Q*) test and *I*^2^ statistic were used to evaluate heterogeneity. An *I*^2^ value of less than 25% was considered indicative of low heterogeneity, in which case a fixed-effects model (FEM) was applied. Conversely, an *I*^2^ value greater than 75% indicated high heterogeneity, warranting the use of a random-effects model (REM). The odds ratio (OR) and 95% confidence interval (CI) were calculated to describe the combined effect sizes, and a forest plot was generated to visually present the results. Sensitivity analysis and funnel plots were used to assess the stability of the results and evaluate potential publication bias. A significance level of *α* = 0.05 (two-sided) was applied.

## Results

### Literature search results

**Figure 1:**
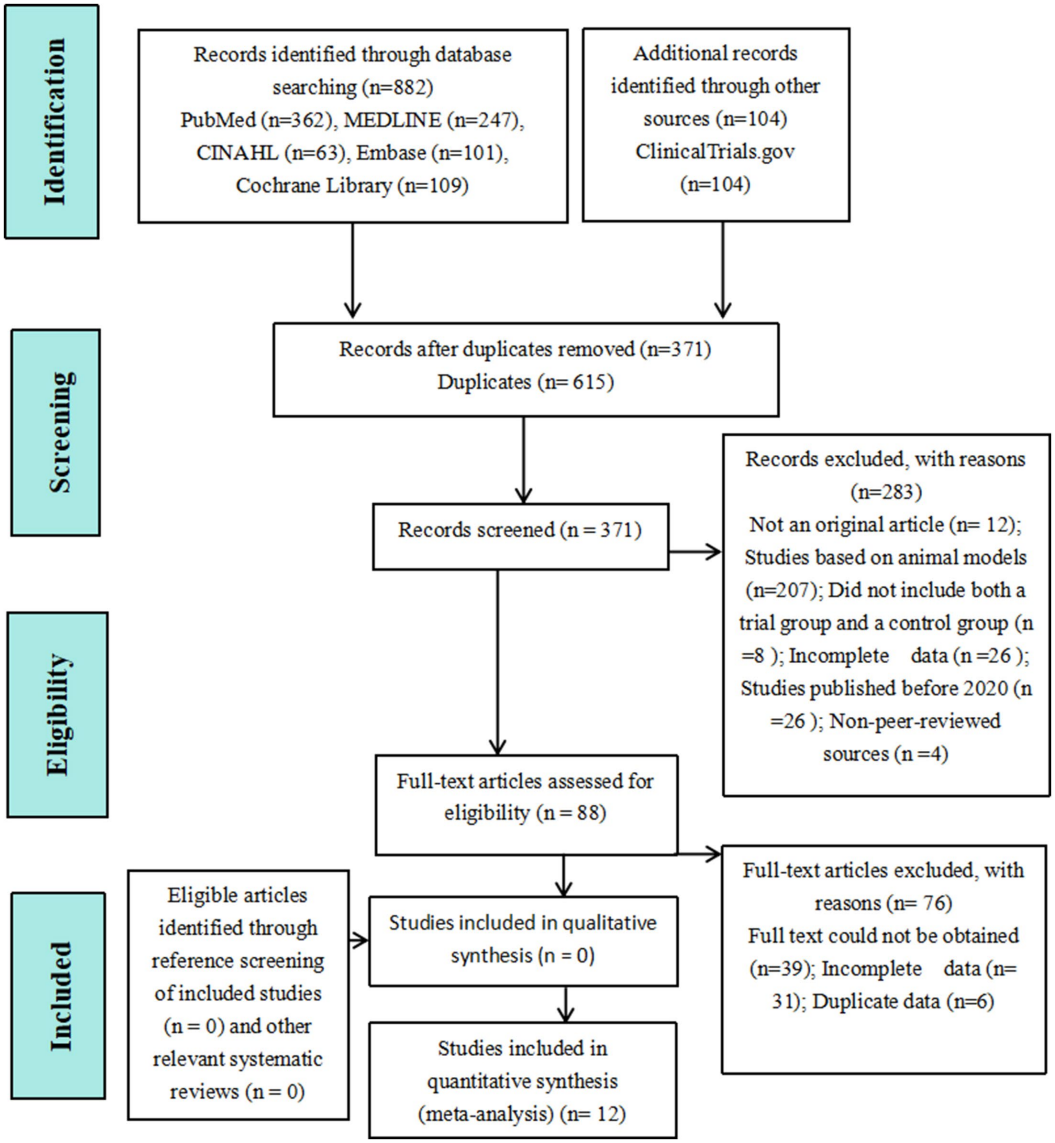
Flowchart of literature search and study selection process.

The literature search initially identified 984 studies, from which duplicates were removed. After screening and full-text review, 12 studies met the inclusion criteria and were included in the analysis. The literature screening process is illustrated in Figure 1.

### Basic characteristics and quality evaluation of literature

The baseline data, including gender, sample size, treatment plans, and outcome indicators, were thoroughly detailed across the 12 included studies. These studies were evaluated using the Cochrane Risk of Bias tool, with the results summarized in Table 1. Regarding intervention characteristics, the included trials applied both manual and mechanical lumbar traction. Procedure duration, treatment frequency, treatment stage, and outcome assessment time points varied across studies and were reported inconsistently. These intervention details, as available, are summarized in Table 1.

**Table 1:** Basic characteristics and quality evaluation of literature.

First author	Year of publication	Sample size		Intervention measure		Outcome index	Judgment
		Experimental group	Control group	Experimental group	Control group		
Chen (24)	2021	38	38	Three steps seven methods massage + traction	Single traction	① ②	Low risk
Zhang (25)	2020	30	30	Kneading − trembling − pushing complex manipulation + traction treatment	Lumbar traction	②	Medium risk
Jin (26)	2020	40	40	Massage + traction	Single traction	①	Medium risk
Wang (14)	2022	100	100	Massage + traction	Single traction	① ② ③ ⑤ ⑥	Medium risk
Li (27)	2022	45	45	Massage + traction	Single traction	① ② ③ ⑤ ⑥	Low risk
Han (28)	2022	36	36	Massage + traction	Lumbar traction	②	Low risk
Liu (15)	2021	100	100	Massage + traction	Single traction	①	Low risk
Cui (29)	2023	115	115	Massage + traction	Lumbar traction	① ② ③	Low risk
Qu (30)	2020	35	35	Massage + traction	Conventional traction	①	Medium risk
Huang (31)	2020	40	40	Massage + traction	Traction	① ④	Medium risk
Jiang (32)	2022	38	37	Massage + traction	Traction	① ② ③	Low risk
Zhu (33)	2022	48	48	Massage + traction	Lumbar traction	① ③ ④	medium risk

① Clinical efficacy; ② JOA score; ③; VAS score; ④ ODI score; ⑤ Lumbar motion score (anterior flexion); ⑥ Lumbar motion score (posterior extension).

### Clinical efficacy

**Figure 2:**
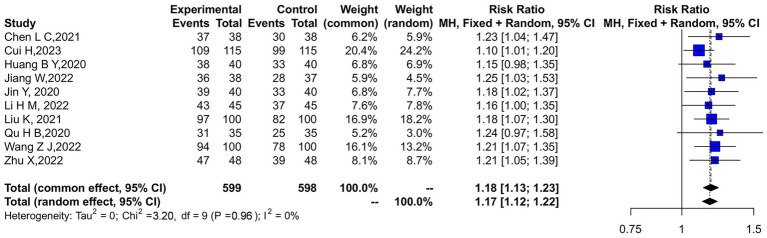
Forest plot of clinical efficacy: a comparison between the experimental and control groups.

Ten studies compared the clinical efficacy between two groups of patients. A heterogeneity test was conducted on the included studies, with results showing *p* = 0.96 and *I*^2^ = 0%. Consequently, a fixed-effects model (FEM) was used to combine the data. The analysis revealed that tuina combined with traction therapy in the experimental group had a significantly better effect on lumbar disc herniation (OR = 4.85, 95% CI: 3.15–7.47, *p* < 0.00001), as illustrated in Figure 2.

### JOA score

**Figure 3:**
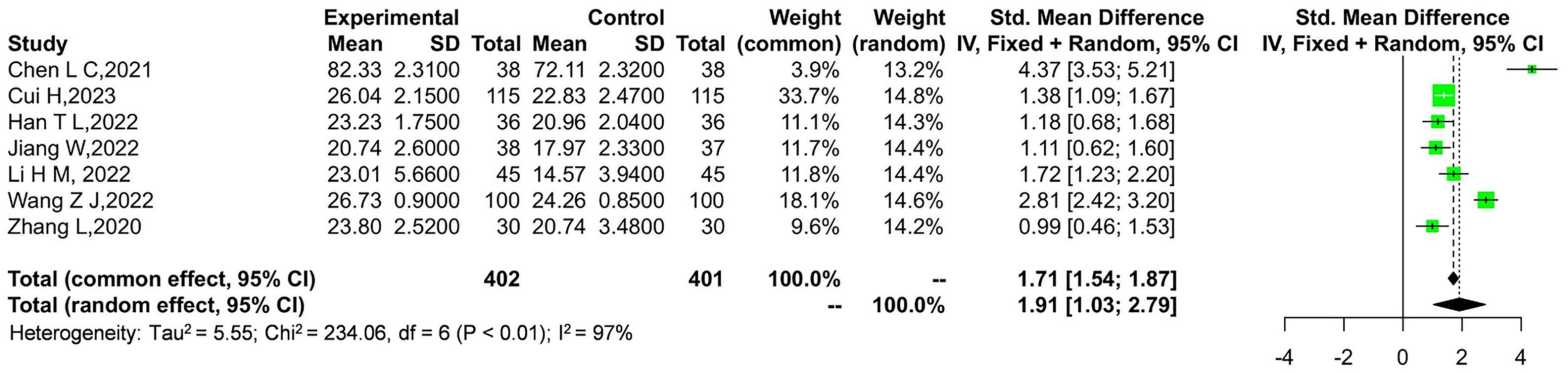
Forest plot of Japanese Orthopaedic Association (JOA) scores comparison between the experimental and control groups.

Seven studies compared the JOA scores between two groups of patients. A heterogeneity test was conducted on the included literature, revealing a *p*-value of <0.00001 and an *I*^2^ value of 97%. Due to the high heterogeneity, a random-effects model (REM) was applied to combine the data. The analysis showed that the treatment method of massage combined with traction in the experimental group resulted in a significantly better JOA score (OR = 4.56, 95% CI: 2.76–6.36, *p* < 0.00001), as illustrated in Figure 3.

**Figure 4:**
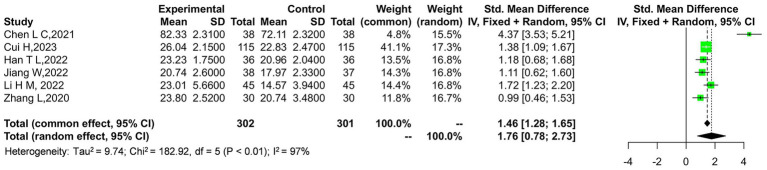
Sensitivity analysis of the JOA scores in the experimental and control groups.

The outcome indicator exhibits considerable heterogeneity, with a Tau-squared value of 9.74 and a chi-square statistic of 182.92 across 5 degrees of freedom, yielding a highly significant *p*-value of 0.00001. This indicates substantial variability among the studies included in the analysis. A sensitivity analysis was conducted by removing the study by Wang et al. (14), which contributed the most weight to the overall effect. The results remained robust, suggesting that the outcome is relatively reliable. As shown in Figure 4, the overall effect still demonstrates a significant difference with a *Z*-score of 3.81, corresponding to a *p*-value of 0.0001, favoring the experimental group over the control.

### VAS score

**Figure 5:**
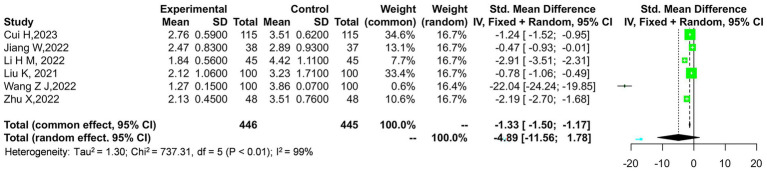
Forest plot of Visual Analogue Scale (VAS) scores comparison between the two groups.

Among the 12 studies, 6 compared the VAS scores between two groups. The heterogeneity test conducted on these studies revealed a *p*-value of <0.00001 and an *I*^2^ value of 99%, indicating high heterogeneity. Consequently, a random-effects model (REM) was used for the combined analysis. The results showed that the experimental group, which received tuina combined with traction, experienced significantly better pain relief (OR = −1.47, 95% CI: −2.40 to −0.55, *p* = 0.0002), as illustrated in Figure 5.

**Figure 6:**
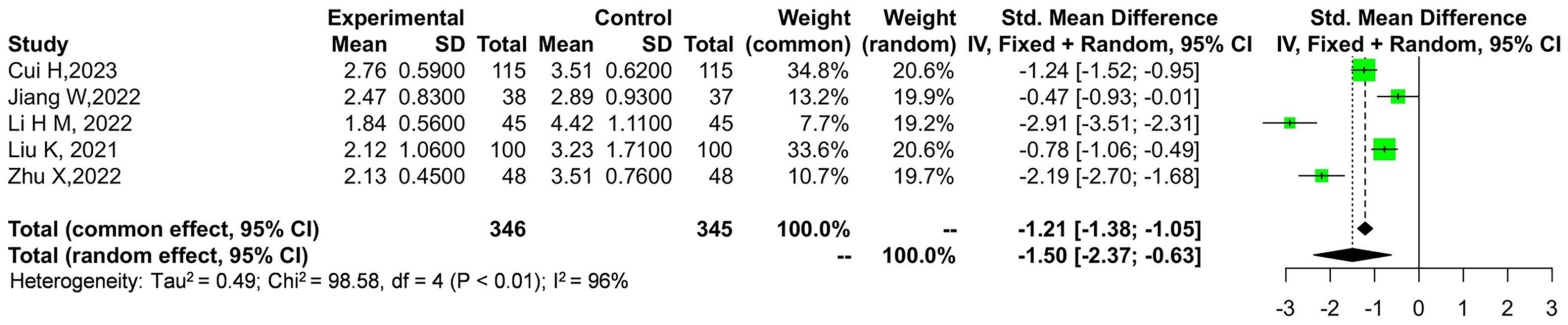
Sensitivity analysis of the VAS scores in the experimental and control groups.

The outcome indicator exhibits significant heterogeneity, with a tau-squared value of 0.49 and a chi-square statistic of 98.58 across 4 degrees of freedom, resulting in a highly significant *p*-value of 0.00001. This level of heterogeneity indicates considerable variability in the study results. To further investigate this, a sensitivity analysis was conducted. Notably, after excluding the study by Liu et al. (15), which contributed the largest weight, the overall effect remained consistent, supporting the reliability of the outcome. The combined effect size across all studies, as shown in Figure 6, reveals a significant mean difference favoring the experimental group over the control, with a pooled mean difference of −1.25 and a 95% confidence interval ranging from −1.88 to −0.62. The overall effect is statistically significant, as indicated by a *Z*-score of 3.88 and a corresponding *p*-value of 0.0001.

### ODI score

**Figure 7:**
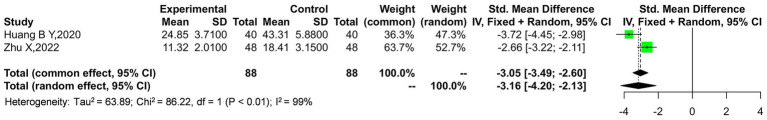
Forest plot of Oswestry Disability Index (ODI) scores comparison between the experimental and control groups.

Among the 12 articles, two compared the primary ODI scores between the two groups. A heterogeneity test was conducted on the included studies, revealing a *p*-value of <0.00001 and an *I*^2^ value of 99%. As a result, a random-effects model (REM) was used for the combined analysis. The findings indicated that the lumbar function in the experimental group, which received tuina combined with traction, was significantly better (OR = −12.73, 95% CI: −23.88 to −1.59, *p* = 0.03), as illustrated in Figure 7.

### Lumbar motion score

#### Forward bending

**Figure 8:**
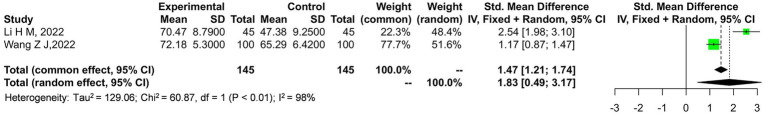
Comparison of forward bending motion scores between the two groups: a forest plot.

Among the 12 articles, 2 compared the forward bending motion scores between the two groups. A heterogeneity test conducted on these studies revealed a *p*-value of <0.00001 and an *I*^2^ value of 98%, indicating substantial heterogeneity. As a result, a random-effects model (REM) was used for the meta-analysis. The results showed no significant difference between the two groups in forward bending motion scores (*p* = 0.07), as illustrated in Figure 8. Although the heterogeneity was high, further sensitivity analysis could not be performed due to the limited number of studies included.

#### Extension

**Figure 9:**
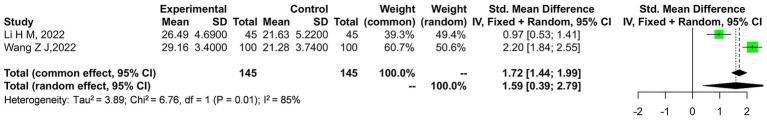
Comparison of post-extension activity scores between the two groups: a forest plot.

Among the 12 studies, 2 compared the lumbar posterior extension range scores between the two groups. A heterogeneity test on the included studies showed a *p*-value of 0.009 and an *I*^2^ value of 95%, indicating significant heterogeneity. Consequently, a random-effects model (REM) was used for the combined analysis. The results demonstrated that the experimental group, which received tuina combined with traction, had significantly better lumbar posterior extension motion scores (OR = 6.51, 95% CI: 3.57–9.46, *p* < 0.0001), as illustrated in Figure 9. Given that this outcome was synthesized from only two studies, these findings are reported as exploratory results and should be interpreted with caution. Although the heterogeneity was substantial, further sensitivity analysis could not be performed due to the limited number of studies included.

### Literature bias examination

**Figure 10:**
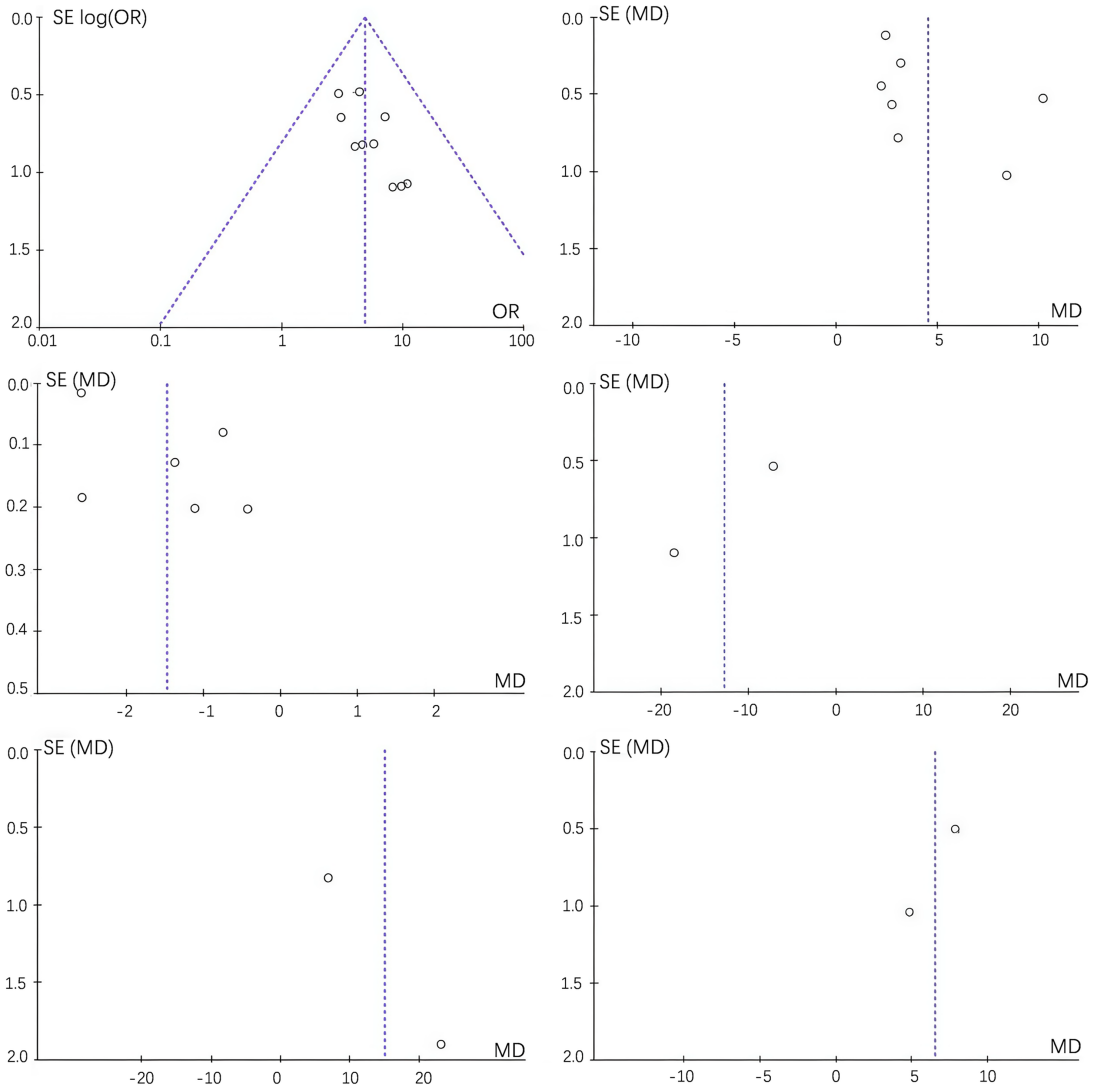
Funnel plot evaluating publication bias between the experimental and control groups.

All outcome indicators were examined for bias, and the results showed asymmetry in the funnel plot, suggesting the presence of bias. Refer to Figure 10.

## Discussion

This meta-analysis integrating 12 randomized controlled trials shows that tuina (Chinese massage) combined with lumbar traction yields superior clinical outcomes compared with traction alone in patients with lumbar disc herniation (LDH). The combined therapy significantly improved overall clinical efficacy (OR = 4.85, 95% CI: 3.15–7.47, *p* < 0.00001), indicating that patients were substantially more likely to achieve a clinically defined response when massage was added to traction. Consistent improvements were also observed in core functional and pain-related indicators, including JOA scores (OR = 4.56, 95% CI: 2.76–6.36, *p* < 0.00001), VAS scores (OR = −1.47, 95% CI: −2.40 to −0.55, *p* = 0.0002), ODI scores (OR = −12.73, 95% CI: −23.88 to −1.59, *p* = 0.03) and lumbar posterior extension scores (OR = 6.51, 95% CI: 3.57–9.46, *p* < 0.0001) (exploratory results based on two studies). Taken together, these results suggest that integrating tuina with traction not only alleviates pain, but also improves lumbar function and daily activity performance in LDH patients.

The findings of this study are broadly consistent with the growing body of literature supporting non-surgical treatments—especially TCM-based therapies—for LDH. Previous systematic reviews and network meta-analyses have indicated that various TCM interventions, such as acupuncture alone or acupuncture combined with manual therapy, can effectively reduce pain and improve function in LDH patients, and in some cases may be comparable to conventional physical therapy or pharmacological approaches [this comparison is mainly discussed in Huang et al. (5) and Qin et al. (12)] (5, 12, 16–19). Within this context, the present meta-analysis helps fill an important evidence gap by focusing specifically on tuina combined with lumbar traction (manual and/or mechanical, as reported in the included trials), rather than acupuncture-based protocols. Our results suggest that, among conservative options, adding tuina to standard lumbar traction may further enhance symptom relief and functional recovery, which is in line with clinical observations that multimodal conservative regimens often outperform single-modality treatments.

The superiority of tuina plus traction over traction alone may reflect complementary biomechanical and neuromodulatory effects. As outlined in the Introduction, lumbar traction may reduce mechanical loading on neural structures and may also influence soft-tissue tension regulation via neuromuscular responses, whereas tuina primarily targets myofascial tissues and pain modulation (10, 11). Tuina, in contrast, focuses on soft tissue and myofascial structures: it can relax paraspinal muscles, reduce muscle spasm, stretch contracted fascia, and improve local microcirculation. Clinically, these effects may extend to deeper myofascial layers by reducing protective muscle guarding and altering neuromuscular reflex activity, which can improve tissue compliance and segmental movement, thereby facilitating functional stabilization during rehabilitation. With respect to swelling and inflammation, tuina is more plausibly linked to modulation of pain-related inflammatory signaling and local tissue perfusion rather than directly reversing the primary mechanical lesion; therefore, the term “attenuate inflammatory responses” in this context refers mainly to secondary, pain-associated inflammatory changes. Importantly, tuina should not be interpreted as directly correcting structural abnormalities (e.g., vertebral subluxation); rather, it may reduce mechanical contributors at the soft-tissue level (such as myofascial stiffness and muscle spasm) and modulate nociceptive processing. When used in combination, traction may address the structural component of nerve compression, whereas tuina may optimize the surrounding soft-tissue environment and pain pathways, thereby producing a synergistic effect that is reflected in the improvements in pain (VAS), function (JOA, ODI) and lumbar posterior extension range observed in this analysis.

An interesting finding of this study is the differential effect of tuina combined with traction on lumbar mobility outcomes. The pooled analysis suggested a significant improvement in posterior extension scores, whereas forward bending motion did not differ significantly between groups; however, these findings should be interpreted as exploratory because each motion direction was reported by only two trials and heterogeneity was substantial (*I*^2^ > 90%). In particular, range-of-motion outcomes are sensitive to variations in assessment procedures (e.g., instructions, end-point definition, examiner technique) and to intervention protocol differences. Traction interventions are known to vary considerably in application parameters and biomechanical effects, which may contribute to direction-specific or inconsistent mobility changes across studies (20–23). Therefore, rather than attributing the observed pattern to specific anatomical structures, we interpret the current evidence as preliminary and emphasize the need for future high-quality trials using standardized traction protocols and standardized range-of-motion assessment to clarify whether mobility improvements differ by movement plane.

Several outcome indicators, particularly JOA, VAS, ODI and lumbar motion scores, displayed high statistical heterogeneity. The variability in traction modality (manual vs. mechanical), treatment duration, and evaluation timing across studies is likely to have contributed to the observed heterogeneity. This heterogeneity likely reflects differences in multiple aspects of study design and clinical practice, including variations in tuina techniques (e.g., specific manipulations, duration and frequency), traction parameters (e.g., force, angle, session length), treatment courses, patient characteristics (age, symptom duration, LDH level and severity) and co-interventions (such as medications or physical modalities). Despite this, sensitivity analyses showed that excluding the most heavily weighted or influential studies using a leave-one-out approach based on study weights in the forest plots did not materially alter the direction or significance of the overall effects on JOA and VAS, as can be verified from the corresponding forest plots. This suggests that the main findings are robust, even though point estimates should be interpreted with caution. In addition, odds ratios were used in parts of the synthesis even though several outcomes are typically measured on a continuous scale, which may reduce the clinical interpretability of the pooled estimates. From a methodological perspective, many included trials were assessed as low or moderate risk of bias, but issues such as inadequate reporting of randomization, lack of blinding, and incomplete description of allocation concealment remain common. These studies were included because they met the prespecified eligibility criteria and addressed the review question; nevertheless, incomplete reporting increases the risk of bias and may exaggerate treatment effects. Accordingly, the pooled findings should be interpreted with caution, and future trials should adopt more rigorous designs and standardized reporting of randomization and allocation concealment.

Funnel plot asymmetry observed in this meta-analysis suggests the possibility of publication bias, with a tendency for positive or statistically significant results to be preferentially published. This phenomenon is common in complementary and alternative medicine research and may lead to overestimation of the true effect size. Furthermore, although this review was originally designed to assess both efficacy and safety, the included studies provided limited and heterogeneous information on adverse events. Accordingly, the absence of reported serious adverse events should not be interpreted as evidence of established safety, but rather as under-reporting of safety outcomes in the available trials. In many RCTs, adverse reactions to tuina and traction were either not reported in detail or were omitted altogether, precluding a meaningful quantitative synthesis of safety outcomes. Therefore, the current evidence is insufficient to establish the safety of tuina combined with traction, and the present meta-analysis cannot definitively characterize their safety profile because adverse events were reported inconsistently or not reported in detail across the included trials. Future trials should adopt standardized adverse event reporting frameworks to better inform risk–benefit assessments.

Despite the above limitations, the present findings have several practical implications. First, for patients with LDH who are not candidates for surgery or who prefer non-surgical options, tuina combined with lumbar traction may be recommended as a potentially more effective alternative to traction alone, particularly for improving pain, functional scores and posterior extension mobility. Second, the evidence supports the concept of multimodal conservative management, where mechanical decompression, soft-tissue regulation and functional rehabilitation are integrated rather than applied in isolation. Third, given the high prevalence of LDH and the substantial burden on quality of life, incorporating standardized tuina-plus-traction protocols into rehabilitation pathways or evidence-based TCM guidelines may help optimize resource allocation and patient outcomes, especially in settings where TCM services are readily available. However, clinicians should individualize treatment decisions based on patient-specific factors, including symptom severity, comorbidities and treatment preferences.

Several limitations of this study should be acknowledged. First, most of the included trials were conducted in a limited geographical and cultural context, which may restrict the generalizability of the findings to other healthcare systems or populations with different biomechanical loading patterns and treatment expectations. Second, sample sizes in individual RCTs were relatively small, and follow-up durations were generally short, making it difficult to assess long-term outcomes such as recurrence, sustained functional improvement, or delayed adverse events. Third, the interventions were heterogeneous, and key parameters of both tuina (specific techniques, practitioner experience, standardization of manipulations) and traction (force, time, frequency) were not consistently reported, which complicates dose–response analysis and protocol optimization. Fourth, most studies focused on subjective or semi-objective outcomes; few incorporated imaging, electrophysiological or biomechanical measurements that could elucidate structural and mechanistic changes. In addition, this review restricted inclusion to studies published from 2020 onward. This decision was made to better reflect contemporary clinical practice, as more recent studies generally employ improved reporting standards, more standardized diagnostic criteria, and clearer descriptions of intervention protocols and outcome measures. However, this restriction may have resulted in the exclusion of earlier relevant studies, and the potential impact of publication time on the pooled estimates cannot be fully excluded. Future reviews could broaden the time frame and perform subgroup or sensitivity analyses based on publication period to further examine the stability of the findings. Future research should address these gaps by conducting large, multicenter RCTs with rigorous methodology, standardized intervention protocols, long-term follow-up, comprehensive safety monitoring and, where feasible, mechanistic sub-studies.

## Conclusion

Patients with lumbar disc herniation who received traditional Chinese massage (tuina) combined with lumbar traction experienced improvements in pain and functional outcomes compared with traction alone. However, these findings should be interpreted cautiously because of substantial heterogeneity across studies and common methodological/reporting limitations (e.g., incomplete reporting of randomization, blinding, and allocation concealment). In addition, mobility-related outcomes reported by only a small number of trials should be considered exploratory. Evidence regarding safety remains insufficient due to limited and inconsistent adverse-event reporting in the included studies. Future well-designed RCTs with standardized traction parameters, consistent outcome assessment time points, and systematic adverse-event reporting are needed to confirm both effectiveness and safety.

## Data Availability

The original contributions presented in the study are included in the article/supplementary material, further inquiries can be directed to the corresponding author.
